# Fructose 1,6-Bisphosphate Aldolase, a Novel Immunogenic Surface Protein on *Listeria* Species

**DOI:** 10.1371/journal.pone.0160544

**Published:** 2016-08-04

**Authors:** Marcelo Mendonça, Gustavo Marçal Schmidt Garcia Moreira, Fabricio Rochedo Conceição, Michael Hust, Karla Sequeira Mendonça, Ângela Nunes Moreira, Rodrigo Correa França, Wladimir Padilha da Silva, Arun K. Bhunia, José Antonio G. Aleixo

**Affiliations:** 1 Laboratório de Imunologia Aplicada, Núcleo de Biotecnologia, Centro de Desenvolvimento Tecnológico, Universidade Federal de Pelotas, Pelotas, RS, Brazil; 2 Molecular Food Microbiology Laboratory, Department of Food Science, Purdue University, West Lafayette, Indiana, United Sates of America; 3 Technische Universität Braunschweig, Institut für Biochemie, Biotechnologie und Bioinformatik, Abteilung Biotechnologie, Spielmannstr, Braunschweig, Germany; 4 Laboratório de Microbiologia de Alimentos, Departamento de Ciência e Tecnologia Agroindustrial, Faculdade de Agronomia Eliseu Maciel, Universidade Federal de Pelotas, Pelotas, RS, Brazil; Universidad Nacional de la Plata, ARGENTINA

## Abstract

*Listeria monocytogenes* is a ubiquitous food-borne pathogen, and its presence in food or production facilities highlights the importance of surveillance. Increased understanding of the surface exposed antigens on *Listeria* would provide potential diagnostic and therapeutic targets. In the present work, using mass spectrometry and genetic cloning, we show that fructose-1,6-bisphosphate aldolase (FBA) class II in *Listeria* species is the antigen target of the previously described mAb-3F8. Western and dot blot assays confirmed that the mAb-3F8 could distinguish all tested *Listeria* species from close-related bacteria. Localization studies indicated that FBA is present in every fraction of *Listeria* cells, including supernatant and the cell wall, setting *Listeria* spp. as one of the few bacteria described to have this protein on their cell surface. Epitope mapping using ORFeome display and a peptide membrane revealed a 14-amino acid peptide as the potential mAb-3F8 epitope. The target epitope in FBA allowed distinguishing *Listeria* spp. from closely-related bacteria, and was identified as part of the active site in the dimeric enzyme. However, its function in cell surface seems not to be host cell adhesion-related. Western and dot blot assays further demonstrated that mAb-3F8 together with anti-InlA mAb-2D12 could differentiate pathogenic from non-pathogenic *Listeria* isolated from artificially contaminated cheese. In summary, we report FBA as a novel immunogenic surface target useful for the detection of *Listeria* genus.

## Introduction

Bacteria of the genus *Listeria* comprise a group of Gram-positive, facultative anaerobic, non-sporulating rods that are widely distributed in the environment [1]. Due to its ubiquitous nature, *Listeria* spp. may persist in food-processing facilities and, thus, may contaminate food products [2]. A short time ago, the genus *Listeria* consisted of eight species; *L*. *monocytogenes*, *L*. *ivanovii*, *L*. *innocua*, *L*. *seeligeri*, *L*. *welshimeri*, *L*. *grayi*, *L*. *marthii*, *L*. *rocourtiae* [3–5]. However, most recently, nine new species have been described: *L*. *fleischmannii*, *L*. *weihenstephanensis*, *L*. *booriae*, *L*. *newyorkensis*, *L*. *floridensis*, *L*. *aquatica*, *L*. *cornellensis*, *L*. *riparia*, and *L*. *grandensis* [6–9]. Among these, *L*. *monocytogenes* is the pathogen that predominantly infects humans resulting in severe infection in the elderly, cancer patients, HIV patients, pregnant women, and their fetuses or infants. Mostly described as an animal pathogen, *L*. *ivanovii* rarely infects human [10]. *Listeria monocytogenes* has been responsible for several fatal outbreaks involving ready-to-eat meat, dairy products (soft cheeses, ice cream), and fish products, and most recently fruits (cantaloupe, apple) and vegetables (celery) [11].

Due to its ubiquitous nature and importance as a food-borne pathogen, countries such as USA have adopted a “zero tolerance” policy for *Listeria* in ready-to-eat (RTE) foods [12]. Other countries, especially those in Europe, have relaxed laws, allowing 100 CFU/25 g for some RTE foods [13], and some countries, such as Brazil, only have rules for production facilities and the bacterium control in high-risk foods, e.g. high-moisture cheeses [14]. Regarding the guidance adopted by each country, the culture-based detection method for *Listeria* relies on the cultivation, isolation, and biochemical characterization of the microorganisms present in the food samples [15,16]. This method is time-consuming, therefore, it represents a drawback for the food production workflow, new and quicker methods, such as the immunochromatographic lateral flow and polymerase chain reaction (PCR), are being developed [17,18].

Antibodies have been widely used in immunological tests for specific detection and identification of pathogens [19–22]. The availability of monoclonal antibodies (mAbs) against bacterial surface antigens not only allows the development of detection assays but also provide a powerful tool for the study of bacterial protein structures and functions [23–27]. Since mAbs recognize exclusive epitopes in the antigen, they can be used to identify new proteins that would be important in the bacterial pathogenesis, survival, or adaptation to the environment [28]. Moreover, mAbs offer a uniform reagent that can be produced in unlimited amounts to provide highly reproducible and consistent immunoassay results [29]. This way, immunoassays were shown to be the best option to overcome the time-consuming method for *Listeria* spp. detection used as standard [15].

Through the years, many groups have focused efforts on the production of mAbs specific to the genus *Listeria* with variable species specificity with unknown or uncharacterized antigen targets [30–33]. At the same time, attempts with variable success were also made to develop *L*. *monocytogenes*-specific antibodies using specific target antigens, such as N-acetyl muramidase [34–36], flagella [37], p60 protein [38,39], Internalin B [40], actin polymerization protein [41], and Internalin A [42]. Although most of these works focused on the detection of the pathogenic species, it is widely known that non-pathogenic *Listeria* grow in a faster rate during the enrichment step, thus possibly increasing false-negative results [43–45]. Thus, the description of new targets that allow the detection of both pathogenic and non-pathogenic species is of high value for the development of detection methods.

Our group had previously described a hybridoma-derived antibody (mAb-3F8) capable of recognizing a 30-kDa protein in all *Listeria* spp. tested. The detection of this protein by mAb-3F8 allowed the specific recognition of *Listeria* genus on the fiber optic immunosensor [42]. In this paper, we show that the 30-kDa protein is a fructose-1,6-bisphosphate aldolase (FBA), an enzyme of the glycolytic pathway that catalyzes the cleavage of its substrate fructose-1,6-bisphosphate (FBP) into glyceraldehyde 3-phosphate (G3P) and dihydroxyacetone phosphate (DHAP). There are two main classes of FBA described: class I is known to form tetramers, and is mainly present in higher eukaryotes, such as animals, plants, and algae; while class II can form many different multimers, and is present mainly in bacteria [46]. Due to a considerable difference between the two classes, class II FBA has been studied as a potential target of new antibiotics [47,48], and as vaccine antigen [49]. Besides, many studies have shown that FBA may play a role in pathogenesis by interacting with host’s plasminogen [50,51], or promoting adhesion to host’s cells [52,53]. Thus, FBA is considered a moonlighting protein (protein with two or more dissimilar functions) in many species [54], and may have significant role in both physiology and pathogenesis.

In addition, here we have also validated the capability of mAb-3F8 to distinguishing different *Listeria* spp., and analyzed FBA’s cellular localization, secretory pathway, and role in mammalian cell adhesion. We additionally showed that mAb-3F8 can be used to detect *Listeria* in contaminated food, as a proof of concept. Moreover, epitope mapping experiments with mAb-3F8 were done to initially characterize this target, highlighting that both FBA and mAb-3F8 are biological tools that can be used to detect *Listeria* genus in food samples.

## Materials and Methods

### Bacterial cultures and growth conditions

The *Listeria* species (Table 1) were grown at 37°C for 16–18 h in Tryptic soy broth (TSB; Becton Dickinson, Sparks, MD, USA) containing 0.6% yeast extract (TSB-YE; Acumedia, Lansing, MI, USA); *Listeria* enrichment broth (LEB, BD); or Fraser Broth (FB, BD). Other non-*Listeria* bacteria were grown in TSB-YE and lactic acid bacteria were grown in MRS broth (DeMan Rogosa Sharpe: BD) at 37°C for 16–18 h.

**Table 1:** Bacterial strains used in this study.

Bacteria / Serotype	Strain/Isolated	Source^a^
***L*. *monocytogenes* 1/2a**	V7	FDA
***L*. *monocytogenes* 1/2b**	F4260	CDC
***L*. *monocytogenes* 1/2c**	7644	ATCC
***L*. *monocytogenes* 4a**	19114	ATCC
***L*. *monocytogenes* 4b**	F4244	CDC
***L*. *monocytogenes* 4c**	19116	ATCC
***L*. *monocytogenes* 4d**	19117	ATCC
***L*. *monocytogenes* 4e**	19118	ATCC
***L*. *monocytogenes* 4ab**	Murray B	FDA
***L*. *monocytogenes* 3a**	19113	ATCC
***L*. *monocytogenes* 3b**	2540	ATCC
***L*. *monocytogenes* 3c**	2479	SLCC
***L*. *monocytogenes* 7**	2482	SLCC
***L*. *innocua***	F4248	CDC
***L*. *innocua* 6a**	Li01	UFPel
***L*. *welshimeri***	35897	ATCC
***L*. *seeligeri***	3954	SLCC
***L*. *seeligeri***	Ls02	UFPel
***L*. *ivanovii***	SE98	USDA
***L*. *grayii***	19120	ATCC
***L*. *marthii***	BAA-1595	ATCC
***L*. *rocourtiae***	109804	CIP
***Bacillus subtilis***	6633	ATCC
***Bacillus thuringiensis***	DUP-6044	MFM-Purdue
***Escherichia coli* O157:H7**	EDL933	CDC
***Lactococcus lactis***	11454	ATCC
***Enterobacter aerogenes***	DUP-14591	MFM-Purdue
***Lactobacillus paracasei***	DUP-13076	MFM-Purdue
***Klebsiella pneumoniae***	---	MFM-Purdue
***Enterococcus faecalis***	---	MFM-Purdue
***Lactococcus lactis* subsp. *Lactis***	HK21	MFM-Purdue
***Enterobacter cloacae***	HK8	MFM-Purdue
***Bacillus cereus***	11778	ATCC
***Staphylococcus aureus***	13301	ATCC
***Pseudomonas aeruginosa***	10145	ATCC
***Salmonella enterica* ser. Typhimurium**	DUP-1167	MFM-Purdue
***Salmonella enterica* ser. Enteritidis**	13076	ATCC

^*a*^ FDA: Food and Drug Administration, Washington, D.C.; CDC: Centers for Disease Control and Prevention, Atlanta, GA.; ATCC: American Type Culture Collection, Rockville, MD.; SLCC: Special *Listeria* Culture Collection, Institute of Hygiene and Microbiology, Univ. of Würzburg, Germany; USDA: National Center for Agricultural Utilization Research, Peoria, Illinois, USA.; MFM-Purdue: Molecular Food Microbiology Lab Collection, Purdue University.; UFPel: Laboratório de Microbiologia de Alimentos Collection, FAEM-UFPel; CIP: Collection de l'Institut Pasteur, Paris, France.

### Protein identification by mass spectrometry

The p30 protein band was first localized in the Coomassie stained SDS-PAGE with the help of the Western blot (mAb-3F8) performed in parallel. The protein band was then excised from the gel and submitted for MALDI-TOF MS+MS/MS (matrix-assisted laser desorption ionization, time-of-flight mass spectrometry followed by tandem mass spectrometry) analysis by two laboratories: Applied Biomics (Hayward, CA, USA) and the Purdue University sequencing facility (West Lafayette, IN, USA). In both laboratories, the Voyager-DE Pro (Applied Biosystems) mass spectrometer was used, and sample treatment and experimental procedures were also the same. Briefly, the gel spots were reduced, alkylated, washed, and dehydrated prior to a trypsin digestion and peptide recovery. After MALDI-TOF, the most abundant peptides were submitted to MS/MS. The data from MALDI-TOF was analyzed by peptide mass fingerprint (PMF), while the data from MS/MS was analyzed by peptide fragmentation mapping. Results from both MS and MS/MS were combined, where tandem MS served as a validation step, and analyzed with MASCOT protein identification software (Matrix Science, London, UK) using three different databases (the NCBI/GenBank, and two of *L*. *monocytogenes* genomes [GenBank: AE017262.2, and FM242711.1]). The hits using the NCBI/GenBank entire database were filtered to those corresponding to *Listeria* proteins. For all three databases, the hits were filtered by including the best protein score of each protein and excluding hits with zero confidence interval (CI%) (S1 Table). The proteins with the highest scores (100 CI%) include, fructose 1,6-bisphosphate aldolase (FBA) class II; transcriptional repressor (CodY); elongation factor Tu (Tuf); and enolase (Eno). Thus, the full protein sequences were obtained from GenBank and analyzed their length/mass and predicted function. This way, it was possible to filter the initial candidates to two hits (*fba* and *codY*) which were further expressed in *E*. *coli*.

### Cloning and expression of recombinant protein target

Specific primers (Eurofins MWG Operon, Germany) were designed to target *fba* and *codY*, (GenBank code: fba: ACK38405.11; codY: CAC99358.1) using Vector NTI 11.0 software (Invitrogen) in order to amplify the complete open reading frame (ORF) of both genes. To insert *fba* and *codY* ORFs into pAE expression vector, the restriction sites for *Bam*HI and *Kpn*I enzymes were incorporated into the forward and reverse primers (For-Fba: 5′-GCGGATCCATGCCTATCGTTAACA-3′; Rev-Fba: 5′-CGGGGTACCTTACGCTTTACCGTTA-3’; For-CodY: 5′-CGGGATCCATGACTTTATTAGAA-3′; Rev-CodY: 5′-CGGGGTACCTTAGTTATTTTTCAA-3’). The ORFs were amplified from the genomic DNA of *L*. *monocytogenes* ATCC 19114 by PCR using an Eppendorf thermocycler (Mastercycler EP gradient S) with the following standardized conditions: 94°C for 7 min, and then 35 cycles consisting of 94°C for 1 min, 50°C for 1 min, 72°C for 2 min, and a final extension of 72°C for 7 min. The amplicons were digested with *Bam*HI and *Kpn*I and ligated into pAE using T4 DNA Ligase (Fermentas, Thermo Fisher Scientific). The pAE-fba and pAE-codY constructs were inserted into *E*. *coli* TOP10 (Invitrogen) by electroporation, and clones were selected on LB-agar containing ampicillin (100 μg/mL). The recombinant plasmids were also transformed into *E*. *coli* BL21 (DE3) pLysS or *E*. *coli* BL21 (DE3) Star competent cells (Invitrogen). The cells were grown until log phase (OD_600_ = 0.5–0.7), when 0.5 mM IPTG was added for 3 h at 37°C. The cultures were then harvested, suspended in lysis buffer (100 mM NaH_2_PO_4_, 10 mM Tris-HCl, and 20 mM imidazole; pH 8.0), and sonicated (5 cycles of 15 s). Then, protein extracts were separated by SDS-PAGE 12%, electrotransferred onto a Hybond-ECL nitrocellulose membrane (GE Healthcare) and immunoprobed with mAb-3F8 and anti-6xHis antibody (Sigma-Aldrich) [55].

### Dot and Western blot assays

*Listeria* spp. were grown in 50 mL of LEB or FB at 37°C for 18 h, centrifuged (7000 ×g, 5 min) and the cell pellets were suspended in the sample solvent (4.6% SDS, 10% β-mercaptoethanol, 0.124 M Tris, and 20% glycerol; pH 6.9). Cells were sonicated four times for 15 s each for total protein preparation. For cell fractionation, crude proteins were prepared from whole cell lysates, and from supernatant, cell wall and cytoplasm as described before [5]. For the Western blot assays, the proteins (~25 μg/well) were separated by sodium dodecyl sulfate-polyacrylamide gel electrophoresis (SDS-PAGE; 10%-acrylamide; Bio-Rad, Hercules, CA, USA) and blotted onto Immobilon-P membranes (Millipore Bedford, MA, USA). The membranes were blocked with 5% skimmed milk for 1 h at room temperature (RT), washed with PBST, and reacted with mAb-3F8 (0.6 mg/mL) and anti-InlA mAb-2D12 (1 mg/mL) diluted in 1:1000 in PBST for 1 h at RT. After washing, the membranes were incubated with an HRP-conjugated goat anti-mouse polyvalent antibody (Sigma, St Louis, MO, USA). Antibody-reactive bands were developed using a chemiluminescence ECL kit (Thermo Fisher Scientific, Rockford, IL, USA) or colorimetric substrate system (6 mg of DAB, 3.3-diaminobenzidine tetrahydrochloride; 10 μL of 30% H_2_O_2_; 9 mL of 50 mM Tris-HCl; pH 7.6; 1 mL of 0.3% nickel sulfate).

Dot blot assays were performed using five microliters of approximately 10^8^ cells/mL of live or heat-killed cell suspensions of *Listeria* spp., *Salmonella enterica* serovar Enteritidis, and *Escherichia coli* O157:H7, spotted onto nitrocellulose membranes (Bio-Rad). The membranes were allowed to air-dry for 15 min, and were then blocked with 5% skimmed milk in PBS for 30 min. Further steps were performed as described above for Western blot.

### Inhibition analysis of *L*. *monocytogenes* adhesion to epithelial cells by mAb-3F8

Freshly grown washed cultures of *L*. *monocytogenes* were suspended in 1 mL of mammalian cell culture medium (DMEM-10F; Dulbecco’s Modified Eagles Medium containing 10% fetal calf serum) to obtain approximately 10^6^ cells/mL. Bacteria were treated with mAb (1 mg/mL) and incubated at 37°C for 20 min with gentle agitation, washed, and suspended in DMEM-10F and added to HCT-8 cell (ileocecal cells; CCL 244; ATCC) monolayer in 24-well plate at a multiplicity of infection (MOI) of 10:1 (bacteria: HCT-8 cell) as before [56]. Non-adherent bacterial cells were removed by washing and the cells were treated with 0.1% Triton X-100 to disrupt cell attachment and bacterial adhesions were quantified by plating serial dilutions onto BHI agar. All experiments were carried out using a positive control anti-InlA mAb-2D12 [42], and a negative anti-N-acetylmuramidase mAb-C11E9 [34].

### Immunoblotting to detect *Listeria* spp. in food samples

The mAb-3F8 and mAb-2D12 [42] were used to identify *Listeria* from food matrices by immunoblotting. *Listeria monocytogenes* ATCC 19114 and *L*. *innocua* (approximately 2 CFU/g) strains were aseptically inoculated into 10 g of Brazilian Minas Frescal cheese (a ready-to-eat soft cheese, Santa Clara brand, purchased from Nacional (Walmart) grocery store in Pelotas, Southern of Rio Grande do Sul state, Brazil). The cheese was then incubated for 15 min at 25°C to promote the interaction of bacteria with the cheese matrix. The samples were then placed in stomacher bags containing 90 mL of FB in each bag, mixed for 2 min using a stomacher (MK 1204, ITR Ltd., Brazil), and incubated at 37°C for 18 h. Cheese samples without inoculated bacteria served as negative controls. Thereafter, 10 mL of each cheese supernatant were collected carefully from the bags to avoid removing any large particles, transferred to 15 mL tubes, and centrifuged (1000 ×g for 10 min). Finally, the pellets were washed twice with PBS-T and suspended in 10 mL PBS. As positive controls, the same *L*. *monocytogenes* and *L*. *innocua* strains were grown in 10 mL of FB and processed as above.

To perform the SDS-PAGE and Western blot, the 10 mL of bacterial suspensions were centrifuged and suspended in 1 mL PBS and transferred to a 1.5 mL tube. After another round of centrifugation, the bacterial pellet was suspended in 100 μL of 2X sample solvent buffer (4.6% SDS, 10% β-mercaptoethanol, 0.124 M Tris, 20% glycerol, pH 6.9) and sonicated (3 times, 15 sec each) and heated for 10 min at 95°C. Samples were separated in SDS-PAGE (12%-acrylamide) and transferred onto PVDF membranes and probed with peroxidase conjugated mAb-3F8 (mAb-3F8-HRP) and mAb-2D12 (mAb-3F8-HRP) and then developed with DAB [42]. The *Listeria* counts from the inoculums and the enrichment broths were determined by plating the cultures on BHI and MOX agar and incubating at 37°C for 24–48 h.

### Construction of an antigen library using ORFeome display technology

The genomic DNA of *L*. *monocytogenes* ATCC 7644 was amplified with *illustra Ready-to-go GenomiPhi V3 DNA amplification kit* (GE Healthcare, Freiburg, Germany) following the manufacturer’s instructions. The amplicon was sonicated with four pulsed cycles of 2 min one pulsed cycle of 1 min. After checking that DNA fragments had between 200 and 1,500 bp in an agarose gel, a DNA-end repair reaction was performed with *Fast DNA End repair kit* (Thermo Scientific, Langenselbold, Germany) as described by the manufacturer. Further, the resulting DNA and cloned into pHORF3 phagemid [57] digested with *Pme*I (New England Biolabs) and transformed into electrocompetent *E*. *coli* SS320 (Lucigen). Transformed cells were titrated and stored at -80°C after overnight growth in a 24.5 cm² dish at 37°C. Afterwards, 1 mL of the *E*. *coli* containing the library was inoculated in 400 mL of 2xYT-GA (2x yeast extract and tryptone medium, containing glucose 100 mM and ampicillin 100 μg/mL) and grown until OD_600_ ≈ 0.5. Twenty-five mL of the culture was transferred to a 50-mL tube and 2.5 × 10^11^ CFU of *Hyperphage* [58] was added. The tube was incubated 30 min at 37°C and 30 min at 37°C under 250 rpm, and then centrifuged (3,220 ×g for 10 min, RT). The pellet was suspended in 10 mL of 2xYT-AK (2xYT containing ampicillin 100 μg/mL and kanamycin 25 μg/mL), and transferred to additional 390 mL of this medium, which was then incubated at 30°C for 24 h. The culture was centrifuged (10,000 ×g, 10 min, 4°C) and the supernatant transferred to another tube in which 50 mL of PEG-NaCl solution (PEG 6,000 20% (w/v), NaCl 2.5 M) was added. The tube was incubated overnight at 4°C and centrifuged (10,000 ×g, 1 h, 4°C), then the pellet was suspended in 10 mL of pre-chilled PBS and centrifuged again (20,000 ×g, 10 min, 4°C). The supernatant was filtered with 0.45-μm membranes and 2.5 mL of PEG-NaCl solution was added. After 20 min on ice, it was centrifuged again (20,000 ×g, 30 min, 4°C), and the pellet suspended in 1 mL PBS. This final suspension containing phage was titrated and had some clones sequenced for library-quality checking.

### Antigen panning for target confirmation

mAb-3F8 was diluted in panning solution (BSA 1% (w/v), dried skim milk 1% (w/v), PBS) and coated on two wells (1.5 μg/well) of a Costar ELISA plate (Corning, Wiesbaden, Germany). Two additional wells were coated with panning solution only; the plate was incubated overnight at 4°C. The *L*. *monocytogenes* ATCC 7644 library (≈1 × 10^10^ CFU/mL) was diluted in panning solution and added to the panning solution well for pre-incubation for 30 min at RT, while the wells containing mAb-3F8 were blocked with the same solution. The library was further transferred to the wells containing the antibody and incubated 1.5 h at RT. Then, the wells were washed and eluted with 10 μg/mL of trypsin diluted in PBS. The eluted phages were used to infect *E*. *coli* TG1 (OD_600_ ≈ 0.5), which was further infected with helperphage M13K07 (MOI 1:20). Infected *E*. *coli* TG1 cells were grown overnight at 30°C, 500 rpm. On the next day, the cultures were centrifuged (3,220 ×g, 10 min, RT) and the supernatant containing phage used instead of the library. In total, three panning rounds were done, and the phage eluted after the 2^nd^ and 3^rd^ rounds were used to infect *E*. *coli* XL1-Blue MRF’, which were diluted, plated on 2xYT-GA agar plates, and grown overnight at 37°C.

### Phage production and ELISA for screening of mAb-3F8 target

The resulting plates from the 2^nd^ and 3^rd^ panning rounds were used to acquire 46 colonies from each, which were transferred to a 96-well culture plate containing 2xYT-GA. The plate was grown overnight at 34°C and then 20 μL was used to inoculate another plate with 180 μL/well of the same medium, which was incubated 2 h at 37°C. Then, clones in each well were infected with *Hyperphage* (MOI 1:20), centrifuged (3,220 ×g, RT, 10 min), and the medium was changed to 2xYT-AK; the plate was then incubated overnight at 30°C, 800 rpm. On the next day, the plate was centrifuged again and 150 μL of the supernatant transferred to another plate, in which 40 μL/well of PEG solution was added and incubated 1 h at 4°C. The plate was centrifuged (1 h, 3,220 ×g, 4°C), the pellet suspended in 150 μL of PBS and centrifuged again (10 min, 3,220 ×g, 4°C). Finally, 50 μL of each supernatant was added to 50 μL of PBS in a Costar ELISA plate (Corning, Wiesbaden, Germany), which was incubated overnight at 4°C for coating. Afterwards, the plate was blocked with 2% MPBS-T (PBS-T with 2% (w/v) of skimmed milk powder) for 30 min at RT, and further incubated with 3F8 antibody (1 μg/mL) for 1 h at RT. Then, goat anti-mouse IgA, M, G HRP-conjugated antibody (AntibodiesOnline, Prod. ABIN376851, 1:4,000) was incubated 1 h at RT. A well with anti-M13 (pVIII) HRP-conjugated (1:40,000) served as positive control for phage production. The reactions were developed with TMB solution for 15 min and read at 450 nm. Reactive clones had their phagemids extracted and sent for sequencing.

### Peptide membrane for the epitope mapping of mAb-3F8

Based on the sequencing results of the screening, a 25-amino acid peptide was identified as the potential mAb-3F8 epitope. To narrow down the amino acids that were important for the interaction, a PepSpots membrane (JPT Peptide Technologies GmbH, Berlin, Germany) was ordered containing 16 peptides with 9 amino-acids each, covering 24 of the 25 amino acids previously identified. The immunoblot was carried out following the manufacturer’s instructions. Briefly, the membrane was activated with methanol, washed with TBS-T, and blocked with 2% MTBS-T overnight at 4°C. Then, mAb-3F8 (1 μg/mL) was added and incubated 3 h at RT, followed by the addition of goat anti-mouse IgA, M, G HRP-conjugated antibody (AntibodiesOnline, 1:4,000), which was incubated 2 h at RT. The membrane was then washed with TBS-T for 1 h at RT prior to the addition of substrate solution (SuperSignal West Pico, Thermo Scientific, Langenselbold, Germany). The images were captured using ChemiDoc XRS equipment (Bio-Rad, München, Germany).

### Sequence and structure analysis of FBA

To access the location of the identified epitope of mAb-3F8, the structure of FBA was predicted using RaptorX online software [59] using standard settings. COTH online software was used to calculate the dimer interfaces for FBA [60]. All structure manipulation and image acquirement were done with PyMol v1.3 software [61]. Multiple sequence alignment of full proteins was done using ClustalOmega tool [55], while alignment of short sequences was done with T-Coffee tool [62]. Phylogenetic analysis were done using ClustalPhylogeny [63]. The identities and similarities were calculated with EMBOSS Needle [64].

## Results

### mAb-3F8-reactive protein is FBA class II

**Fig 1:**
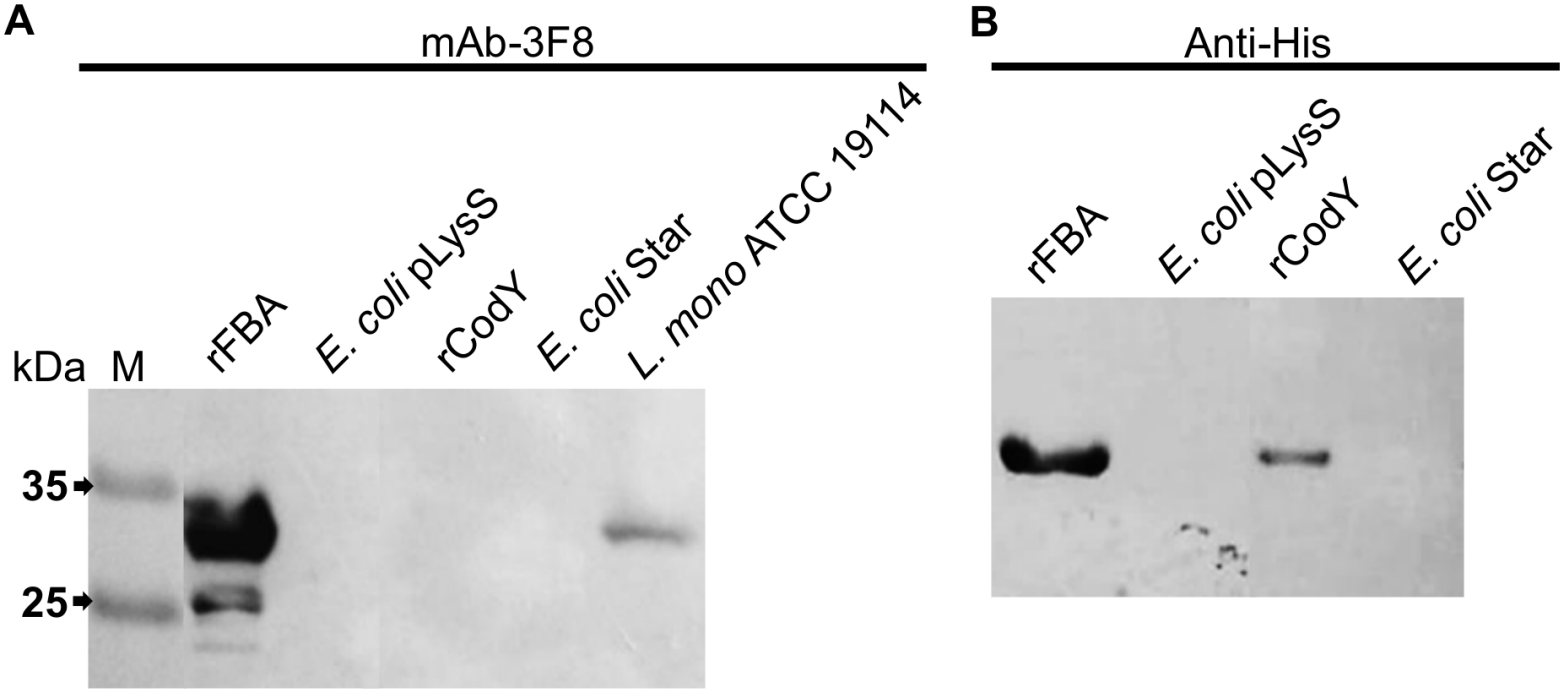
Western blot assay with recombinant rCodY and rFBA proteins to determine the target of mAb-3F8. **(A)** Western blot using mAb-3F8 as primary antibody shows that this mAb reacts with purified rFBA, as well as protein extract of *L*. *monocytogenes*, but not with rCodY. **(B)** The reaction using Anti-His primary antibody as control shows that both rFBA and rCodY were present in detectable amounts on the membrane.

Following the MALDI-TOF mass spectrometry analysis, four proteins—fructose 1,6-bisphosphate aldolase (FBA) class II, transcriptional repressor CodY, elongation factor Tu (Tuf), and enolase (Eno)–received the highest scores with 100 CI %, and thus were further analyzed using Swiss-Prot and NCBI databases. FBA is a 284-amino-acid (aa)-long protein with a calculated MW of 29,936 Da, and is close to our SDS-PAGE-estimated molecular mass (30-kDa) (Fig 1). Thus, mAb-3F8-reactive 30-kDa protein was presumably considered the FBA class II (lmo2556). CodY (lmo1280) is a 259-aa-long intracellular GTP binding protein with an approximate MW of 28,614 Da [65] and is primarily located in the intracellular compartment, and mAb-3F8 reacts with whole cells, thus CodY was less likely to be the target, but it was cloned to validate our results. Tuf is a 395-aa-long protein with estimated mass of 43 kDa [66], which is much higher than the expected 30 kDa. Likewise, Eno is a 430-aa-long protein with estimated mass of 46 kDa [67] and this size is well above 30 kDa. Hence, these last two proteins were excluded from being a potential target of mAb-3F8.

Next, we cloned both *fba* (encoding FBA) and *codY* (encoding CodY) into *E*. *coli* expression vectors to produce rFBA and rCodY proteins and to confirm their reactivity with the mAb-3F8. Both recombinant proteins were affinity purified and after immunoblotting, only rFBA showed strong reactions with mAb-3F8 while rCodY did not (Fig 1A). Anti-6xHis antibody was used as a positive control to the assay (Fig 1B). This confirms that the antigen for mAb-3F8 is FBA class II protein.

### FBA is detectable only in *Listeria* species

**Fig 2:**
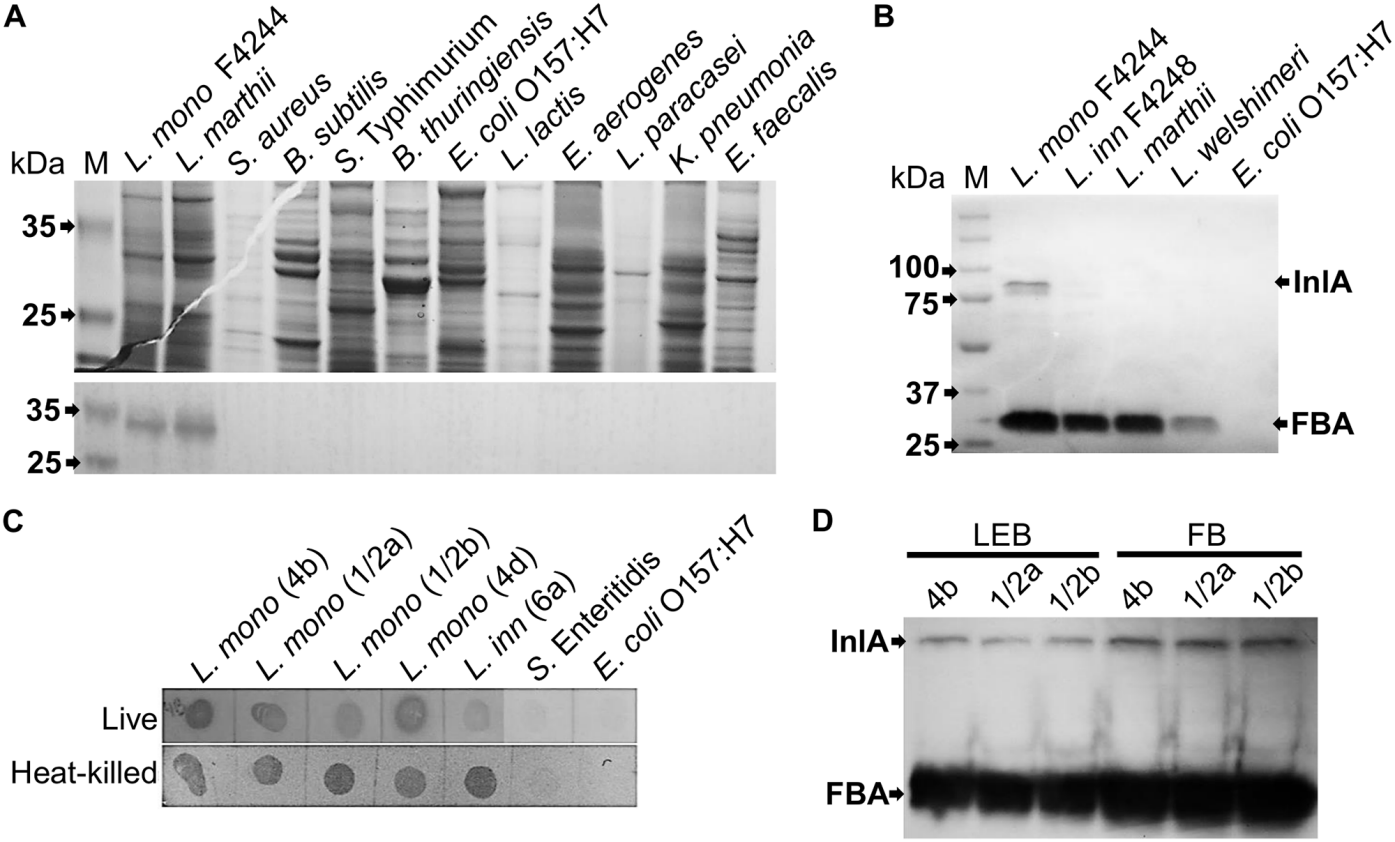
Western and dot blots showing the presence of FBA in *Listeria* spp. only. **(A)** SDS-PAGE 12% showing the protein preparations of two *Listeria* species and many related bacteria (upper part). In Western blot using mAb-3F8, only those extracts from *Listeria* showed a 30-kDa band corresponding to FBA. **(B)** Western blot using mAb-3F8 showing the presence of FBA in other *Listeria* species, but not in *E*. *coli* O157:H7 used as negative control. In this blot, mAb-2D12 (anti-InlA) was used together with mAb-3F8, showing an 88-kDa band corresponding to InlA, which is present only in *L*. *monocytogenes*. **(C)** The results found in the Western blot were same as the dot blot, in which only *Listeria* spp. (either live or heat-killed) could be detected when using mAb-3F8. **(D)** Western blot using both mAb-3F8 and -2D12 allows detection of three different serotypes of *L*. *monocytogenes* (4b, 1/2a, and 1/2b), no matter the medium used for their growth (LEB or FB).

In our previous work, we have shown the specific reaction of mAb-3F8 with a single protein band of about 30 kDa present in the eight *Listeria* species tested and the 13 serotypes of *L*. *monocytogenes* [42]. In the current work, we showed again that the mAb-3F8 had no cross-reaction with additional Gram-positive or Gram-negative bacteria tested in Western blot (*Staphylococcus aureus*, *Bacillus subtilis*, *B*. *cereus*, *B*. *thuringiensis*, *Salmonella enterica* ser. Typhimurium, *S*. *enterica* ser. Enteritidis, *E*. *coli* O157:H7, *Lactococcus lactis*, *Enterococcus aerogenes*, *Lactobacillus paracasei*, *Klebsiella pneumonia*, and *Enterococcus faecalis*) (Fig 2A). It is also shown that the target is recognized specifically in *Listeria* species both in Western blot (Fig 2B), and dot blot using both live and heat-killed cells (Fig 2C). Since it is a non-described target for *Listeria* spp., we also tested its expression pattern in the two most common selective media (LEB, FB) used to isolate the pathogen. After a Western blot with whole cell lysates of the 3 most frequent serotypes of *L*. *monocytogenes* (4b, 1/2a, and 1/2b), it is clear that FBA is expressed regardless the medium used (Fig 2D).

### FBA is present in all cellular fractions and supernatant, but its secretion is not SecA2-dependent and it does not play role in adhesion

**Fig 3:**
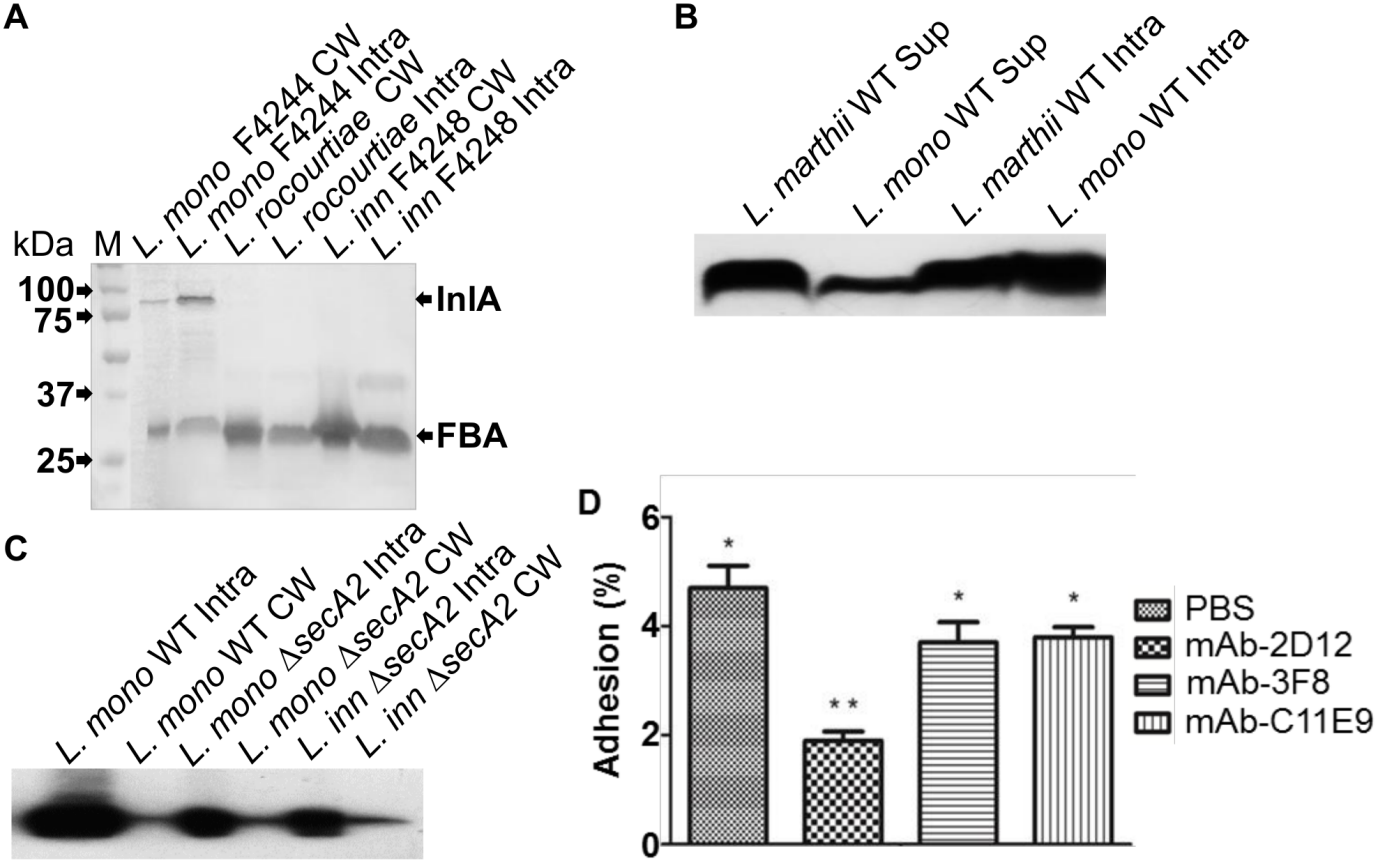
Cell localization and adhesion experiments for FBA characterization. **(A)** Western blot using mAb-3F8 show that FBA protein is present in the cell wall and intracellular (membrane and cytoplasm) of three different *Listeria* species tested. The top band shows reaction with anti-InlA mAb-2D12. **(B)** When using only mAb-3F8 in Western blot, it is possible to observe that FBA is also present in the culture supernatant of two different *Listeria* species. **(C)** Due to the presence of FBA in the cell wall, a *L*. *monocytogenes* mutant (Δ*secA2*) was used to inquire the secretion pathway of FBA. However, no difference in band intensity was observed in the cell wall (CW) or intracellular (Intra) fractions between the wild type (WT) and mutant strains, indicating that the secretory pathway of FBA is not SecA2-dependent. **(D)** Adhesion experiments *in vitro* using HCT-8 cells show that mAb-3F8 has no inhibition activity, since it’s pre-incubation with *L*. *monocytogenes* cells shows similar levels of adhered bacteria as the negative controls with PBS and mAb-C11E9. The positive control mAb-2D12 (anti-InlA) was the only one to show significant reduction in adhesion.

The analysis of *Listeria* spp. cellular fractions in Western blot revealed that FBA protein is present in all cellular fractions, i.e. cell wall, and intracellular (cytoplasm and membrane) (Fig 3A). Additionally, to the cell wall and intracellular fractions, Western blot with the secreted proteins from *L*. *monocytogenes* and *L*. *marthii* was made and showed that the protein is found in the culture supernatant (Fig 3B). Since translocation of many *Listeria* proteins to cell surface and the extracellular milieu (i.e., secretion) is aided by SecA2, an auxiliary secretion system, we examined the reaction of mAb-3F8 with protein preparations from cell wall and intracellular fractions of Δ*secA2* deletion mutant of *L*. *monocytogenes* and *L*. *innocua* strains [5]. However, there was no difference in reaction of mAb-3F8 to proteins from WT or Δ*secA2* deletion strains in neither fractions, suggesting that SecA2 pathway is not essential for the translocation of FBA protein to the cell wall or membrane (Fig 3C).

We also examined if FBA is involved in *L*. *monocytogenes* adhesion to mammalian cells. Adhesion experiment using the human ileocecal cell line, HCT-8, indicated that pretreatment of *L*. *monocytogenes* with mAb-3F8 did not block *L*. *monocytogenes* adherence, indicating that it is probably not involved in adhesion. In contrast, anti-InlA mAb-2D12 used as positive control significantly (*P* < 0.05) reduced adhesion of this bacterium (Fig 3D), since InlA is a well-known adhesion and invasion factor [68]. A negative control antibody, mAb-C11E9 that reacts with N-acetylmuramidase, had no effect on the adhesion of *L*. *monocytogenes* as expected [34,69]. These data indicate that FBA may not play a role in adhesion of *L*. *monocytogenes* on human intestine.

### FBA allows the detection of *L*. *monocytogenes* from food

**Fig 4:**
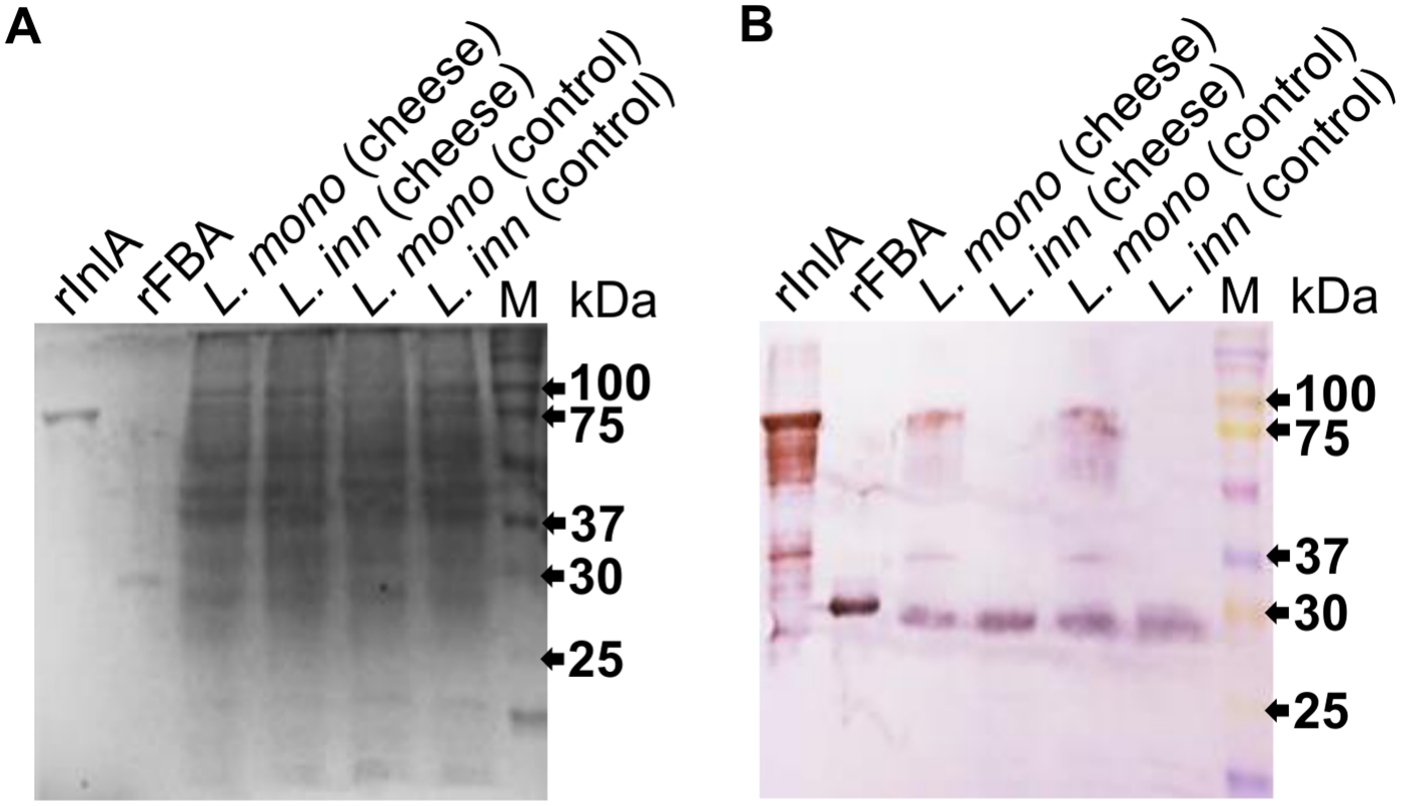
SDS-PAGE and Western blot of bacteria from artificially contaminated cheese. **(A)** SDS-PAGE (12%-acrylamide) showing the protein extracts from the bacteria after enrichment step using stomacher bags. Protein extracts from pure cultures were used as control. **(B)** Western blot using both mAb-3F8 and -2D12 on these protein extracts shows the expected detection bands: both InlA (88 kDa) and FBA (30 kDa) for the pathogenic strain; and only FBA (30 kDa) for the non-pathogenic. Purified rInlA and rFBA, and the pure cultures of *L*. *monocytogenes* and *L*. *innocua* were used as positive control for the antibodies.

Western blot analysis of the cheese samples inoculated with *L*. *monocytogenes* allowed the detection of the two analyzed proteins: InlA (≈88 kDa), and FBA (≈30 kDa). Thus, it confirms the presence of *L*. *monocytogenes* in the sample. The cheese sample inoculated with *L*. *innocua* indicated the presence of a single band, corresponding to the 30-kDa FBA as expected. The bands in the protein extracts of the strains had the same size of purified proteins used as control in the assay (Fig 4). The cells isolated from the cheese were plated on MOX plates, indicating the presence of *Listeria* spp. in inoculated samples.

### mAb-3F8 epitope allows distinguishing *Listeria* spp. in sequence level and is part of the catalytic site of FBA dimer

**Fig 5:**
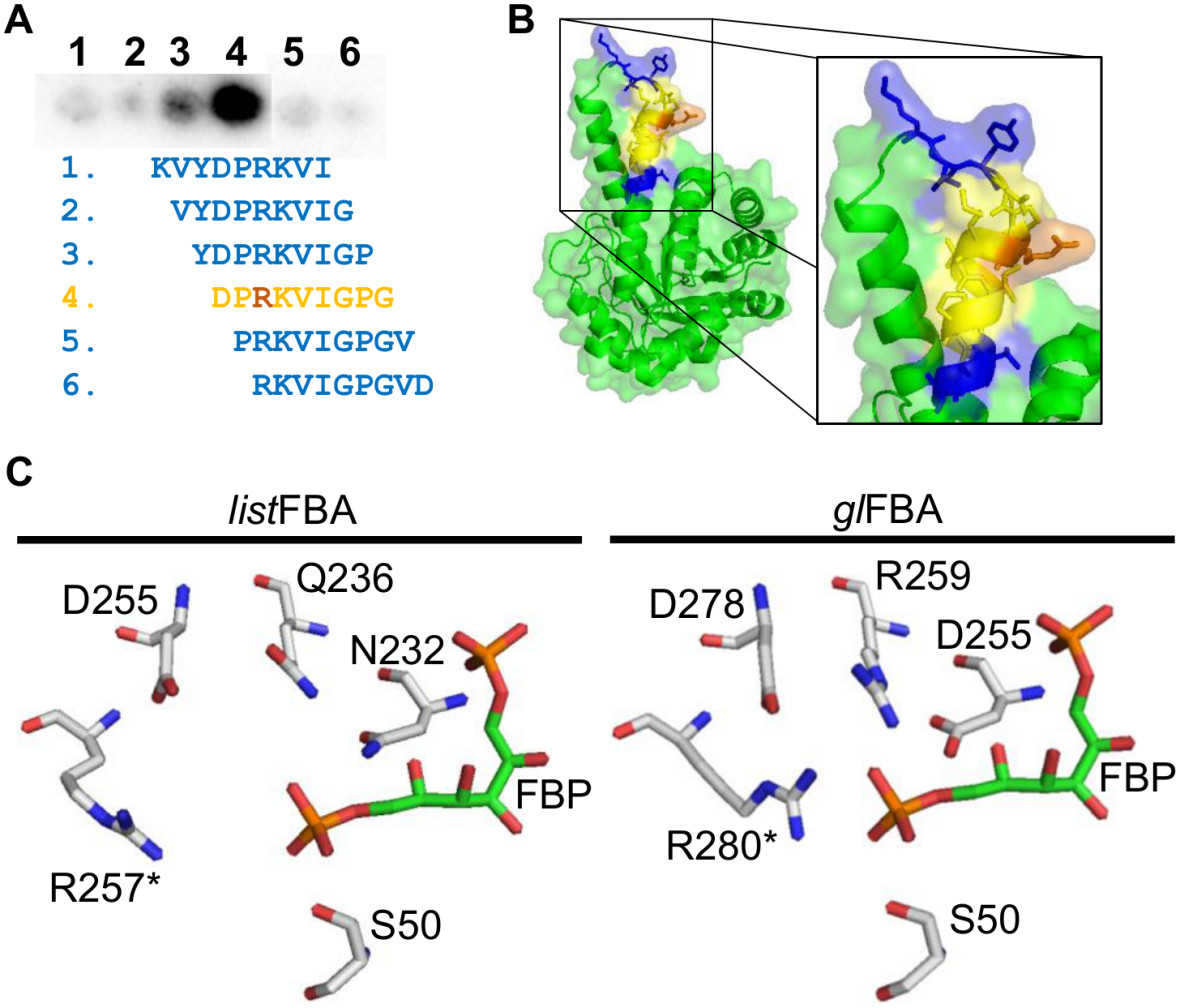
Epitope mapping of mAb-3F8 and position of the epitope in FBA structure. **(A)** By using a peptide membrane, which was incubated with mAb-3F8 as primary antibody, it was possible to identify a 14-amino acid sequence as the epitope of mAb-3F8. In this sequence, it is also possible to observe that a 9-amino acid region (yellow) gives a higher reaction and, thus, is likely the most important recognition part of the epitope. Interestingly, Arg257 (orange), which may be part of the catalytic site of the dimeric form of FBA is present in the epitope. **(B)** The position of the epitope in FBA structure shows it is a loop-to-helix transition, which has many amino acid side chains exposed on the protein surface. **(C)** A closer look on the G3P site (part of the catalytic site) shows that Arg257 of *Listeria* spp. FBA (*list*FBA, left part) can play role on binding G3P, since binding pocket is similar to that from *Giardia lamblia* FBA (*gl*FBA, right part). For graphical reasons, FBP molecule fitting in *list*FBA pocket has the same position of that one from *gl*FBA, thus it may not represent the real fitting of this molecule in the structure. Asterisk (*) indicate that the amino acid is present in the partner subunit of the protein dimer.

An antigen library of *L*. *monocytogenes* was built using ORFeome display technology. By employing this library against mAb-3F8, it was possible to detect a 25-aminoacid peptide from FBA that was possibly the epitope of the antibody. To narrow down the exact sequence involved in the recognition, a peptide membrane was used, in which it was possible to define a 14-amino acid region as the epitope (Fig 5A). We also noticed that a 9-amino acid sequence contributes mostly to the interaction with the antibody. When aligning FBA sequences from all organisms tested in Western blot, we observed that those from *Listeria* spp. are isolated in the cladogram, though it is closer to *Bacillus* species, and *S*. *aureus* (S1A Fig). An interesting finding is that the 14-amino acid peptide identified as mAb-3F8 epitope gives almost the same effect on the cladogram (S1B Fig). In fact, these findings are in accordance with the identity between the analyzed proteins, which show that only *Bacillus* spp. and *S*. *aureus* have >50% identity with *Listeria* spp. when considering the identified epitope (S2 Table).

After predicting the structure of FBA with RaptorX software, it was possible to observe that the epitope sequence represents a transition from a loop to α-helix in FBA’s structure (Fig 5B). Since the prediction was made using FBA from *Bacillus anthracis* (PDB code: 3Q94) as template that has no publication describing the structure and no detailed information regarding this structure. However, when comparing the predicted structure with that from *Giardia lamblia* (*gl*FBA, PDB code: 3GAY), it is likely that the epitope is part of the catalytic domain within the dimeric form of the protein. This part is called G3P site—referring that it can bind the G3P molecule—and is similar between the two proteins (Fig 5C). In this site, Arg257 (Arg280 in *gl*FBA) of the partner subunit, which is present in the identified epitope, may play a role in the interaction with carbon 6 of FBP substrate.

## Discussion

FBA, an enzyme present in both prokaryotes and eukaryotes, where FBA class I is associated with animals and plants, and class II with bacteria, archae, and unicellular eukaryotes [70]. FBA class II catalyzes the reaction of fructose-1,6-bisphosphate to D-glyceraldehyde-3-phosphate and dihydroxyacetone phosphate, and its reverse reaction, steps that are important for the glycolysis and gluconeogenesis. Since it has many other functions besides the enzymatic activity, FBA is considered a “moonlighting” enzyme and seem to be crucial for the viability of either Gram-positive or Gram-negative bacteria, since its knockout turns many species unviable [71].

In the present study, we show that FBA class II from *Listeria* spp. is mainly a cytoplasmic or membrane-associated protein. In agreement with our result, FBA was described to be present in both membrane and cytosolic fractions of *L*. *monocytogenes* as determined by proteomic analysis [72]. Moreover, our results also show that this protein can be detected in the cell wall of *Listeria* spp., allowing the already described detection of the whole cells, as well as in the supernatant [42]. Likewise, FBA was also found in the cell wall of *Streptococcus pneumoniae* [73], and in the cell wall and the supernatant fraction of *Mycobacterium tuberculosis* [50]. This protein is present in *Neisseria meningitides*, but was not detected in the supernatant fraction [53]. Additionally, FBA was shown to be one of the most abundant soluble proteins of *E*. *coli* with more than 47 thousand protein copies per cell [74]. In accordance to these data, our Western blot results suggest that this protein is abundantly expressed in the *Listeria* species tested as well.

The “moonlighting” protein FBA is present in many species, and it is known that this kind of proteins can be involved in adhesion to host’s cells, binding to host’s proteins, or even the modulation of the immune response [54]. In *N*. *meningitidis*, FBA is highly conserved and participates in the adhesion of this bacterium to human cells [53]. Likewise, FBA from *S*. *pneumoniae* also provides adhesion to the host’s cells [52]. FBA from *Mycobacterium tuberculosis* has been shown to bind human plasminogen suggesting an involvement of this enzyme in host-pathogen interactions [50]. Similarly, FBA from *Paracoccidioides* spp. also binds to plasminogen and is likely to contribute to pathogenesis [51]. Although FBA function as a “moonlighting” protein is described in the literature for many pathogens, little is known about this protein in *Listeria* genus. In the present study, we detected no role on adhesion when using HCT-8 cells, practically ruling out this FBA function in *Listeria* spp., although other cell types should be further tested.

FBA is essential for the metabolism of many microorganisms, therefore, its sequence and structure tends to be conserved among the same species, and even between different ones. This characteristic has been explored to design broad-spectrum vaccines against *Streptococcus pneumoniae*, being able to protect mice against respiratory challenges of different strains [49,75]. In another study, FBA from *Candida albicans* was used as diagnostic tool in an indirect detection of *Candida* spp. infections, showing 87.1 and 92.8% of sensitivity and specificity, respectively [76]. Following the principle that FBA allows broad-spectrum detection, we describe this enzyme as a target for direct detection of the food-borne bacteria from *Listeria* genus. This is the first time FBA is described as a biomarker for *Listeria* spp., and can open new possibilities for the improvement on the detection of this pathogen both in food and in clinical samples.

Nevertheless, the fact that FBA is structurally conserved in different species is interesting, especially because mAb-3F8 specifically recognizes FBA from *Listeria* spp., but not other closely related bacteria. Thus, strategies for epitope mapping of mAb-3F8 were performed in order to better understand the principle of the detection. By using pHORF phage display technology [77–80], we were able to build an ORFeome library and find a very short sequence (25 amino acids) that contained the epitope, which was studied in detail by using a peptide membrane. This way, we could find an epitope with 14 amino acids that shares considerable identity (78.6) and similarity (85.7) only with *Bacillus* spp., when considering the species tested in Western blot. *Bacillus* spp. could not be recognized by mAb-3F8 in Western blot, therefore, we assume that the 3 amino acid-difference between the 2 proteins—one of them is in the 9-amino acid part that seems to be the most important for recognition—are enough to eliminate the binding properties of mAb-3F8 (see S1 and S2 Texts). Interestingly, the identified epitope is part of the catalytic site of the protein in its dimeric form. Thus, we show that an antibody can be targeted to this protein in *Listeria* spp. and possibly block its activity via binding to the active site. Considering that FBA is also studied as a target for antibiotic treatment [47,48], the use of mAbs like 3F8 in therapy could be an intriguing possibility, but impairment of the enzymatic activity should be verified.

As a proof of concept, FBA can be used as a biomarker to identify *Listeria* spp. directly from contaminated food. Cheese was spiked with *L*. *monocytogenes*, which was further enriched and detected using mAb-3F8 in Western blot. Using this mAb alone, it was possible to detect both *L*. *monocytogenes* and *L*. *innocua*. When combining mAb-3F8 and mAb-2D12, which recognizes InlA present only in *L*. *monocytogenes*, it was possible to distinguish the pathogenic species from the non-pathogenic one. The principle of distinguishing non-pathogenic and pathogenic *Listeria* relies on the possible overgrow of the former, which can impair the detection of the latter [44,45]. Although the detection of non-pathogenic species only means the food is safe for consumption, it also indicates that the food production or storage environment is prone to the persistence of the bacteria, including the pathogenic species. Thus, detecting non-pathogenic species is important indicator for the requirement of improved sanitary and hygienic practices during food production.

## Conclusions

In many countries, *L*. *monocytogenes* is a major public health concern and always a potential economic burden regarding food industry. Besides, the increasing discovery of new species in different environmental locations increases the importance of studying this bacterium. Here we describe for the first time that FBA is a surface protein of *Listeria* spp., and that mAb-3F8 against this protein can specifically recognize the genus via a conserved epitope. Thus, both mAb-3F8 and FBA have great potential as immunodiagnostic tools to detect *Listeria* spp. and, if combined with another antibody, distinguish the pathogenic species. Thus, mAb-3F8 can be further explored in an effort to improve immunoassays for *Listeria* detection. Additionally, this work gives first descriptions of properties of *Listeria* FBA, as well as describes mAb-3F8 as an analytical tool to investigate the role of this enzyme in *Listeria* spp. physiology or pathogenesis.

## Supporting Information

S1 Fig Phylogenetic tree of FBA and the corresponding mAb-3F8 epitope of all bacteria used in Western blot. (A) Despite being a conserved protein, FBA sequence allows distinguishing *Listeria* genus from other closely related species, such as *Bacillus* spp. and *S*. *aureus*. (B) The same distinction is observed when only the epitope of FBA, which is recognized by mAb-3F8, is used for the alignment. Thus, this sequence can in principle distinguish *Listeria* spp. already in sequence level.
(TIF)

S1 Table Protein hits of MALDI-TOF MS+MS/MS after filtering the results from MASCOT with three different databases. (DOCX)

S2 Table GenBank codes, identity and similarity of the FBA sequences from the organisms used in immunoblots. (DOCX)

S1 Text Alignment of the FBA sequences from the species used in Western blots with mAb-3F8. (DOCX)

S2 Text Alignment of the epitope of mAb-3F8 with the corresponding sequences of FBA from the species used in Western blots. (DOCX)
