# Supplementary figures and images for: Fructose 1,6-Bisphosphate Aldolase, a Novel Immunogenic Surface Protein on Listeria Species
Source: PLoS One. 2016 Aug 4;11(8):e0160544. doi: 10.1371/journal.pone.0160544 (PMC4973958; doi:10.1371/journal.pone.0160544)

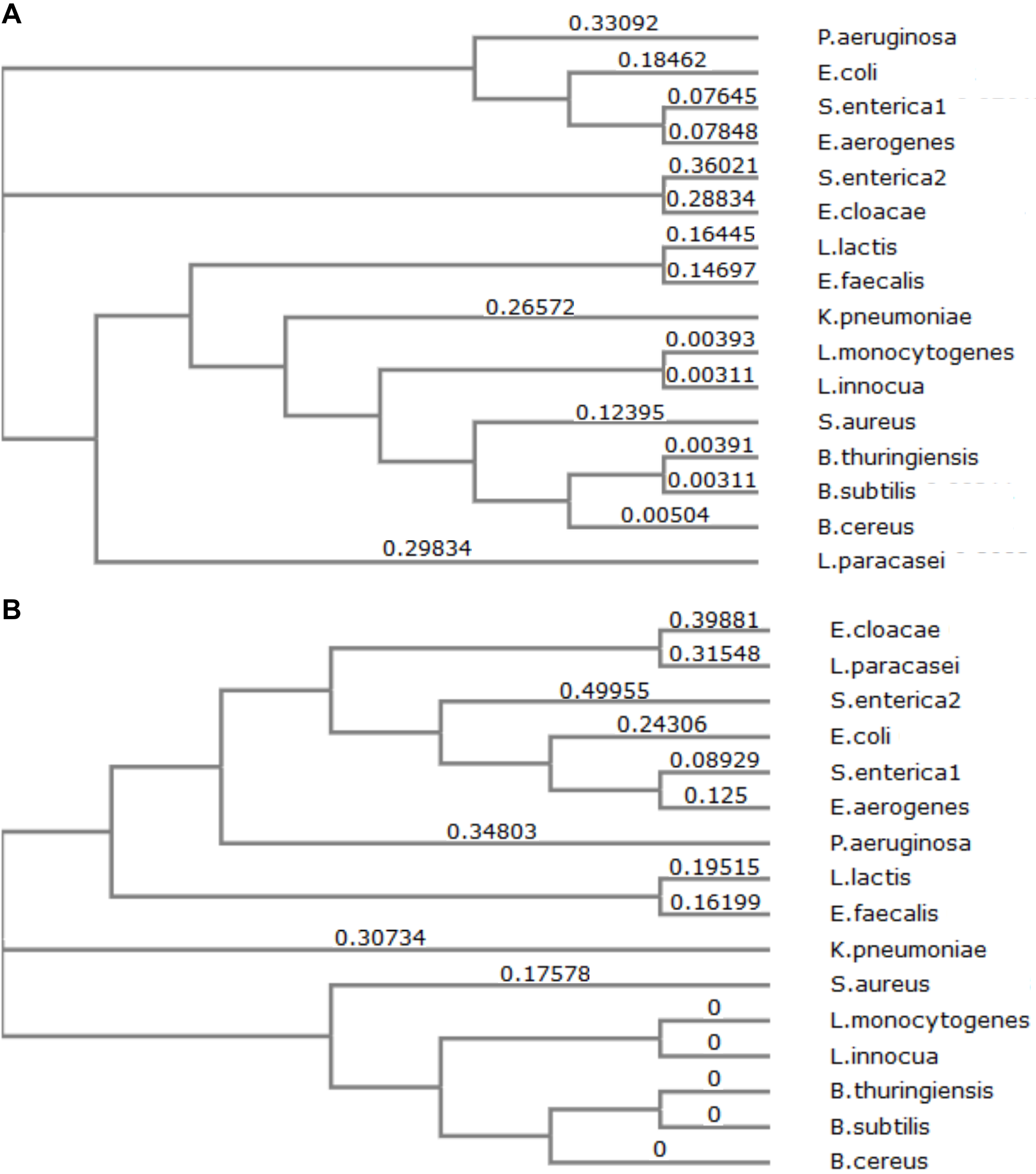

Supplement: S1 Fig — (A) Despite being a conserved protein, FBA sequence allows distinguishing Listeria genus from other closely related species, such as Bacillus spp. and S. aureus. (B) The same distinction is observed when only the epitope of FBA, which is recognized by mAb-3F8, is used for the alignment. Thus, this sequence can in principle distinguish Listeria spp. already in sequence level. (TIF) [file pone.0160544.s001.tif]
