# Supplementary material for: Fructose 1,6-Bisphosphate Aldolase, a Novel Immunogenic Surface Protein on Listeria Species
Source: PLoS One. 2016 Aug 4;11(8):e0160544. doi: 10.1371/journal.pone.0160544 (PMC4973958; doi:10.1371/journal.pone.0160544)
Supplement: S1 Table — (DOCX) [file pone.0160544.s002.docx]

**S1 Table**. Protein hits of MALDI-TOF MS+MS/MS after filtering the results from MASCOT with three different databases

| NCBI/GenBank Database | Protein hit | Accession no. NCBI | Protein score | Protein score CI %^a^ | Ion score | Ion score CI %^a^ |
| --- | --- | --- | --- | --- | --- | --- |
| NCBI | Enolase | WP_003727923.1 | 225 | 100 | 194 | 100 |
|  | Fructose-1,6-bisphosphate aldolase | NP_466079.1 | 209 | 100 | 186 | 100 |
|  | Transcriptional repressor CodY^b^ | ZP_05289370.1 | 177 | 100 | 160 | 100 |
|  |  |  |  |  |  |  |
| *L. monocytogenes* F2365 | Enolase | WP_003726579.1 | 207 | 100 | 194 | 100 |
|  | Fructose-1,6-bisphosphate aldolase | WP_003724024.1 | 199 | 100 | 186 | 100 |
|  | Elongation factor Tu | WP_003723640.1 | 129 | 100 | 111 | 100 |
|  | L-lactate dehydrogenase | WP_003722739.1 | 57 | 99.475 | 52 | 99.999 |
|  | DNA gyrase subunit A | WP_003725619.1 | 55 | 99.167 | ND | ND |
|  | Hypothetical protein | WP_003724915.1 | 53 | 98.520 | ND | ND |
|  | Hypothetical protein | WP_003724719.1 | 52 | 98.179 | ND | ND |
|  | UvrABC system protein C | WP_010958872.1 | 51 | 97.654 | ND | ND |
|  | Hypothetical protein | WP_003728005.1 | 49 | 96.608 | ND | ND |
|  | Aamino acid ABC transporter, substrate-binding protein | WP_003728238.1 | 48 | 95.922 | ND | ND |
|  | Mmolecular chaperone DnaK | WP_003726023.1 | 42 | 82.201 | 22 | 99.430 |
|  | Hhypothetical protein | WP_003727523.1 | 41 | 76.536 | ND | ND |
|  | Hypothetical protein | WP_003726138.1 | 39 | 62.812 | ND | ND |
|  |  |  |  |  |  |  |
| *L. monocytogenes* Clip80459 | Enolase | WP_003726579.1 | 207 | 100 | 194 | 100 |
|  | Fructose-1,6-bisphosphate aldolase | WP_003724024.1 | 199 | 100 | 186 | 100 |
|  | Elongation factor Tu | WP_003723640.1 | 129 | 100 | 111 | 100 |
|  | L-lactate dehydrogenase | WP_003722739.1 | 57 | 99.485 | 52 | 99.999 |
|  | DNA gyrase subunit A | WP_003725619.1 | 55 | 99.184 | ND | ND |
|  | UvrABC system protein C | WP_003726544.1 | 51 | 97.699 | ND | ND |
|  | Hypothetical protein | WP_003728005.1 | 49 | 96.675 | ND | ND |
|  | Amino acid ABC transporter, substrate-binding protein | WP_003728238.1 | 48 | 96.002 | ND | ND |
|  | Hypothetical protein | WP_003728005.1 | 48 | 95.081 | ND | ND |
|  | Molecular chaperone DnaK | WP_003726023.1 | 42 | 82.548 | 22 | 99.424 |
|  | Hypothetical protein | WP_003727523.1 | 41 | 76.993 | ND | ND |
|  | Hypothetical protein | WP_003726138.1 | 39 | 63.537 | ND | ND |
|  | MerR family transcriptional regulator | WP_012681243.1 | 37 | 42.210 | ND | ND |

^a^Confidence interval percentage, or “CI %”.

^b^The transcriptional repressor CodY was only detected by Applied Biomics company, and was not present as possible hit when analyzed at the Purdue University Proteomics facility.

ND, not determined due to low abundancy in the first MS analysis.
