# Supplementary material for: Fructose 1,6-Bisphosphate Aldolase, a Novel Immunogenic Surface Protein on Listeria Species
Source: PLoS One. 2016 Aug 4;11(8):e0160544. doi: 10.1371/journal.pone.0160544 (PMC4973958; doi:10.1371/journal.pone.0160544)
Supplement: S2 Table — (DOCX) [file pone.0160544.s003.docx]

**S2 Table**. GenBank codes, identity and similarity of the FBA sequences from the organisms used in immunoblots

| Organism | GenBank code | Identity (%)^a^ | Similarity (%)^a^ | Identity (%)^b^ | Similarity (%)^b^ |
| --- | --- | --- | --- | --- | --- |
| *Listeria monocytogenes*^c^ | ACK38405.1 | 100 | 100 | 100 | 100 |
| *L. innocua* | WP_003768394.1 | 99.3 | 100 | 100 | 100 |
| *Bacillus cereus* | WP_001131851.1 | 69.8 | 80.7 | 78.6 | 85.7 |
| *B. subtili* | CUB52089.1 | 69.8 | 80.7 | 78.6 | 85.7 |
| *B. thuringiensis* | WP_042969964.1 | 70.2 | 80.7 | 78.6 | 85.7 |
| *Lactococcus lactis* | WP_038602049.1 | 48.3 | 65.7 | 57.1 | 78.6 |
| *Staphylococcus aureus* | WP_001662541.1 | 65.6 | 78.6 | 57.1 | 78.6 |
| *Klebsiella pneumonia* | WP_048334153.1 | 50.5 | 67.2 | 57.1 | 64.3 |
| *Enterococcus faecalis* | WP_010818782.1 | 51.5 | 68.4 | 50.0 | 71.4 |
| *Pseudomonas aeruginosa* | KWX43640.1 | 34.6 | 52.4 | 35.7 | 50.0 |
| *Salmonella enterica* 1 | WP_000469981.1 | 39.7 | 57.5 | 35.7 | 42.9 |
| *Enterobacter aerogenes* | WP_020077858.1 | 38.1 | 54.0 | 28.6 | 35.7 |
| *Lactobacillus paracasei* | AKU58293.1 | 46.3 | 63.6 | 21.4 | 50.0 |
| *Escherichia coli* | WP_000289782.1 | 36.7 | 52.4 | 21.4 | 35.7 |
| *Enterobacter cloacae* | WP_023299105.1 | 41.5 | 58.2 | 20.0 | 46.7 |
| *Salmonella enterica* 2 | WP_001131801.1 | 30.3 | 53.1 | 3.8 | 3.8 |

^a^ Identity and similarity corresponding to the whole FBA sequence.

^b^ Identity and similarity corresponding to the 14-amino acid sequence found as the epitope.

^c^ *L. monocytogenes* sequence was used as reference in the alignment.
