# Supplementary material for: Fructose 1,6-Bisphosphate Aldolase, a Novel Immunogenic Surface Protein on Listeria Species
Source: PLoS One. 2016 Aug 4;11(8):e0160544. doi: 10.1371/journal.pone.0160544 (PMC4973958; doi:10.1371/journal.pone.0160544)
Supplement: S1 Text — (DOCX) [file pone.0160544.s004.docx]

**S1 Text**. Alignment of the FBA sequences from the species used in Western blots with mAb-3F8.

P.aeruginosa ---MALISMRQMLDHAAEFGYGVPAFNVNNLEQMRAIMEAADKTDSPVIVQASAGARKYA

S.enterica2 ---MPLVNGRILLNCIQEKHVLAGAFNTTNLETTISILNAIERSGLPNFIQIAPTNAQL-

E.coli ---MYVVSTKQMLNNAQRGGYAVPAFNIHNLETMQVVVETAANLHAPVIIAGTPGTFTHA

S.enterica1 ---MFIISSKNMLQKAQHAGYAVPAFNIHNLETLQVVVETAAEMRSPLIVAGTPGTFSYA

E.aerogenes ---MYIISSKNMLLKAQRHGYAVPAFNIHNLETMQVVVETAAELRSPLILAGTPGTYSYA

E.cloacae ---MPLISLADGLAHAREHRYALGAFNVLDSHFLRALFAAAKQERSPFIINIAEVHFKYV

L.lactis ---MAIVSAEKFVQAARDNGYAIGGFNTNNLEWTQAILRAAEAKKTPVLIQTSMGAAKYM

E.faecalis ---MPVVSGAEFLKAARKGGYAVGGYNTNNLEWTQAILEAAEAKKAPVLIQTSMGAAKYM

L.paracasei MGKMALVSATSLVASALADRYAIGHFNINGADWLETYLKVAQTTKTPIIVATSDRIIDFL

K.pneumoniae ----MLVSMKDMLQHALRDGYAVGQFNINNLEWVGAVLSTAQQCRSPVILGVSGGTVKHM

L.monocytogenes ---MPIVNMTDMLKKALAGKYAVGQFNINNLEWTQAILKAAEAEKAPVILGVSEGAAKYM

L.innocua ---MPIVNMTDMLKKALAGKYAVGQFNINNLEWTQAILKAAEAEKSPVILGVSEGAAKYM

S.aureus ---MPLVSMKEVLIDAKENGYAVGQYNINNLEFTQAILEASQEENAPVILGVSEGAARYM

B.thuringiensis ---MPLVSMKEMLNKALEGKYAVGQFNMNNLEWTQAILAAAEEEKSPVILGVSEGAARHM

B.subtilis ---MPLVSMKEMLNKALEGKYAVGQFNMNNLEWTQAILAAAEEEKSPVILGVSEGAARHM

B.cereus ---MPLVSMKEMLNKALEGKYAVGQFNMNNLEWTQAILAAAEEEKSPVILGVSEGAARHM

::. : :* . . . * :: :

P.aeruginosa GAPFLCHLILAA--IEEFPHIPVVMHQDHGTSPDVCQRSIQLGFSSVMMDGSLREDGKTP

S.enterica2 SGYDYIYEIVKR--HADKMDIPVSLHLDHGKTLEDVKQAVRAGFTSVMIDGAAL------

E.coli GTENLLALV-SA--MAKHYHHPLAIHLDHHTKFDDIAQKVRSGVRSVMXDASHL------

S.enterica1 GMGNIVAIA-GD--LAREYNLPLAIHLDHHESLADIESKVMAGIRSVMIDGSHF------

E.aerogenes GTGNVVAIA-RD--LAKIWDLPLALHLDHHEDLADITHKVQAGIRSVMIDGSHS------

E.cloacae SLESLV---EAVKFEAARHDIPVVLNLDHGLHFEAVVRALRLGFSSVMFDGSTL------

L.lactis GGYKMCKLLIETLVESMGITVPVAIHLDHGH-FDDALECIEVGYSSLMFDGSHL------

E.faecalis GGYKVAKDMITNLVDSMNITVPVAIHLDHGD-YEAALECIEVGYTSIMFDGSHL------

L.paracasei GGFDYMARYVRFMMQALSVTVPVALHLDHGLSVDHVYQAIDAGYTSVMFDGSKL------

K.pneumoniae LGLKCIHDIVVNAMEYLRIDVPVALHLDHGTTREACEAAIAAGFSSIMFDGSHL------

L.monocytogenes GGFKTVVKMTEGLVEDLKITVPVAIHLDHGSSFDSCKAAIDAGFSSVMIDGSHH------

L.innocua GGFKTVVKMTEGLVEDLKITVPVAIHLDHGSSFDSCKAAIDAGFSSVMIDGSHH------

S.aureus SGFYTIVKMVEGLMHDLNITIPVAIHLDHGSSFEKCKEAIDAGFTSVMIDASHS------

B.thuringiensis TGFKTVVAMVKALIEEMNITVPVAIHLDHGSSFEKCKEAIDAGFTSVMIDTSHH------

B.subtilis TGFKTVVAMVKALIEEMNITVPVAIHLDHGSSFEKCKEAIDAGFTSVMIDASHH------

B.cereus TGFKTVVAMVKALIEEMNITVPVAIHLDHGSSFEKCKEAIDAGFTSVMIDASHH------

*: :. ** : * *:* * :

P.aeruginosa ADYDYNVRVTQQTVAFAHACGVSVEGELGCLGSLETGMA-GEEDGVGAEGVLDHSQLLTD

S.enterica2 -PLEENIAFTREAVDFCKSFGVPVEAELGAILGKEDDHVSE-------------ADCKTE

E.coli -PFAQNISRVKEVVDFCHRFDVSVEAELGQLGGQEDDVQ-VNE----------ADAFYTN

S.enterica1 -PFEENVALVKSVVDFCHRYDTSVEAELGRLGGIEDDLV-VDS----------KDALYTN

E.aerogenes -PFEENVALVKNVVALSHRYDASVEAELGRLGGVEDDLV-VDA----------KDALYTN

E.cloacae -SYEENIRQTREVVKMCHAVGVSVEAELGAVGGDEGGALYGHA----------DETFFTD

L.lactis -PIEENLKLAEEVIAKAHAKGISVECEVGSIGGEEDGIV-G-------------EGELAP

E.faecalis -PFEENLKLAKDVVEKAHAKGISVECEVGSIGGEEDGII-G-------------TGELAD

L.paracasei -PIGENVALTKEVVAYAHAHHVSVEAEVGSVGGNENGLV-N-------------GIRYAS

K.pneumoniae -PFRENLAITRHLVTLAHSKGISVEAELGTIAGSEDGIVNS-------------EVIYAD

L.monocytogenes -PIDENIAMTKQVVDYAHAKGVSVEAEIGTVGGDEDGVT-G-------------GINYAD

L.innocua -PIDENIAMTKQVVDYAHAKGVSVEAEIGTVGGDEDGVT-G-------------GINYAD

S.aureus -PFEENVATTKKVVEYAHEKGVSVEAELGTVGGQEDDVV-A-------------GIIYAD

B.thuringiensis -PFEENVETTKQVVEYAHARNVSVEAELGTVGGQEDDVIAE-------------GVIYAD

B.subtilis -PFEENVETTKKVVEYAHARNVSVEAELGTVGGQEDDVIAE-------------GVIYAD

B.cereus -PFEENVETTKKVVEYAHARNVSVEAELGTVGGQEDDVIAE-------------GVIYAD

*: .. : .: ** *:* : . * :

P.aeruginosa PEEAADFVKKTKVDALAIAIGTSHGAYKFTKPPTGDTLSIQRIKEI-----HARIPDTHL

S.enterica2 PEKVQRFVEETGCDMLAVSIGNVHGLE--DIPR----IDIPLLQRI------ASVSPVPL

E.coli PAQAREFAEATGIDSLAVAIGTAHGMYASA-PA----LDFSRLENI------RQWVNLPL

S.enterica1 PQQARAFVARTGIDSLAVAIGTAHGMYAAE-PK----LDFERLAEI------RALVDIPL

E.aerogenes PEQAREFVARTGIDSLAVAIGTAHGLYTAE-PK----LDFDRLAAI------RGCVDVPL

E.cloacae PQLAREFVDSTGIDALAVAIGNAHGKYKGE-PK----LDFPRLDAI------RQQTGLPL

L.lactis IDDAVAM-AKLGVDFLAAGIGNIHGPYPENWKG----LHIDHLEKLNKALVEAMGHNVPI

E.faecalis IEECKQM-VATGIDYLACGIGNIHGQYPENWKG----LAFDHLQAI----AEAVGSDVPL

L.paracasei VADAVEM-ASTGIDALAAALGSVHGDYVGR-PN----LNFERMAEI------AAATKLPL

K.pneumoniae PQECYTLVTETQVDCLAAALGSTHGLYKGK-AR----LGFSEMKAI------AERVKVPL

L.monocytogenes PQECLRVVKEANIDALAAALGSVHGPYHGE-PV----LGFDEMKEI------SELTGAPL

L.innocua PQECLRVVKEANIDALAAALGSVHGPYHGE-PV----LGFDEMKEI------SELTGAPL

S.aureus PKECQELVEKTGIDALAPALGSVHGPYKGE-PK----LGFKEMEEI------GLSTGLPL

B.thuringiensis PAECKHLVEATGIDCLAPALGSVHGPYKGE-PN----LGFAEMEQV------RDFTGVPL

B.subtilis PAECKHLVEATGIDCLAPALGSVHGPYKGE-PN----LGFAEMEQV------RDFTGVPL

B.cereus PAECKHLVEATGIDCLAPALGSVHGPYKGE-PN----LGFAEMEQV------RDFTGVPL

. * ** .:*. ** : : : : :

P.aeruginosa VMHGSSSVPQDWLAIINEYGGEIKETYGVPVEEIVEGIKYGVRKVNIDTDLRLASTGAIR

S.enterica2 VIHGGSGI---------------------DADILRSFVNYKVAKVNIASDLRKAFITTVG

E.coli VLHGASGL---------------------STKDIQQTIKLGICKINVATELKNAFSQALK

S.enterica1 VLHGASGL---------------------PESDIRQAISLGVCKVNVATELKIAFSDALK

E.aerogenes VLHGASGL---------------------PDSDIRRAISLGVCKVNVATELKIAFSDALK

E.cloacae VLHGGSGI---------------------SDTDFRRAIELGIHKINFYTGMSQAALAAVE

L.lactis VLHGGSGI---------------------PDDQIKEAIANGVAKVNVNTECQLAFAAATR

E.faecalis VLHGGSGI---------------------PQEQIEKAISMGISKVNVNTEFQLSFAKATR

L.paracasei VLHGASGI---------------------LDDQIQQAIQLGTAKININTEVNTVWTSAVT

K.pneumoniae VLHGGTGI---------------------ADEDMRRAIACGTAKINVNTENMYAWCQQVK

L.monocytogenes VLHGGSGI---------------------PEHQIKKAIELGHSKINVNTECQIVWTAAVR

L.innocua VLHGGSGI---------------------PEHQIKKAIELGHSKINVNTECQIVWTAAVR

S.aureus VLHGGTGI---------------------PTKDIQKAIPFGTAKINVNTENQIASAKAVR

B.thuringiensis VLHGGTGI---------------------PTADIEKAISLGTSKINVNTENQIEFTKAVR

B.subtilis VLHGGTGI---------------------PTADIEKAISLGTSKINVNTENQIEFTKAVR

B.cereus VLHGGTGI---------------------PTADIVKAISLGTSKINVNTENQIEFTKAVR

*:**.:.: : : *:*. :

P.aeruginosa RFLAQ---------NPSEFDPRK-YFSKTVEAMRDICIARYEAFGTAGNA-------

S.enterica2 KAWVNN---------NNEA-NLARVMANAKQAVEDDVYSKIKMMNKNHSAFRKVS--

E.coli NYLTE---------HPEATDPRD-YLQSAKSAMRDVVSKVIADCGCEGRA-------

S.enterica1 EYFLQ---------NPKANDPRH-YMQPAKQAMKEVVRKVIHVCGCEGQL-------

E.aerogenes TYFLE---------NPGANDPRH-YMTPAKAAMKEVVRKVIHVCGCEGQL-------

E.cloacae QRMAN---------RQSLYDEFAELLLGIEEAITDTVAEQMRIFGSAGQA-------

L.lactis KFVNEFDANEAEYMKKKLFDPRK-FLKPGFDAITASVEERIDVFGSANKA-------

E.faecalis EYIEAG-----KDLEGKGFDPRK-LLAPGKTAIIKDAEEHIDWFGSANKA-------

L.paracasei KALQA-------K--RSNHDPQP-ILTAGKQAIAYLVEAKMKAFHVLGKSTRLSSMS

K.pneumoniae AIFAA-------DTGHDVNDPRK-VIAQGLQPVREMIARRMALFGSQQRY-------

L.monocytogenes EKLAT-------D--DKVYDPRK-VIGPGVDAIIKTVSEKIQEFGSNGKA-------

L.innocua EKLAT-------D--DKVYDPRK-VIGPGVDAIIKTVTEKIQEFGSNGKA-------

S.aureus DVLNN-------D--KEVYDPRK-YLGPAREAIKETVKGKIKEFGTSNRAK------

B.thuringiensis EVLNK-------D--QEVYDPRK-FIGPGRDAIKATVIGKIREFGSNGKA-------

B.subtilis EVLNK-------D--QEVYDPRK-FIGPGRDAIKATVIGKIREFGSNGKA-------

B.cereus EVLNK-------D--QEVYDPRK-FIGPGRDAIKATVAGKIREFGSNGKA-------

: :
