# Supplementary material for: Fructose 1,6-Bisphosphate Aldolase, a Novel Immunogenic Surface Protein on Listeria Species
Source: PLoS One. 2016 Aug 4;11(8):e0160544. doi: 10.1371/journal.pone.0160544 (PMC4973958; doi:10.1371/journal.pone.0160544)
Supplement: S2 Text — (DOCX) [file pone.0160544.s005.docx]

**S2 Text**. Alignment of the epitope of mAb-3F8 with the corresponding sequences of FBA from the species used in Western blots.

E.cloacae SLYDEFAELLLGIEE

S.enterica2 NEANLARVMANAKQ-

L.paracasei SNHDPQPILTAGKQ-

E.coli EATDPRDYLQSAKS-

S.enterica1 KANDPRHYMQPAKQ-

E.aerogenes GANDPRHYMTPAKA-

P.aeruginosa SEFDPRKYFSKTVE-

L.lactis KLFDPRKFLKPGFD-

E.faecalis KGFDPRKLLAPGKT-

K.pneumoniae DVNDPRKVIAQGLQ-

S.aureus EVYDPRKYLGPARE-

L.monocytogenes KVYDPRKVIGPGVD-

L.innocua KVYDPRKVIGPGVD-

B.thuringiensis EVYDPRKFIGPGRD-

B.subtilis EVYDPRKFIGPGRD-

B.cereus EVYDPRKFIGPGRD-

: :
